# Audiological Profile and Factors Associated With Hearing Impairment Among Adults With Allergic Rhinitis in Tamil Nadu, India: A Hospital-Based Cross-Sectional Study

**DOI:** 10.7759/cureus.115705

**Published:** 2026-09-03

**Authors:** Harish Vel V., Neethu Sara George, Arun Kumar G., Neeraj V Mohandas, Thanisha I.

**Affiliations:** 1 Otorhinolaryngology, Arunai Medical College and Hospital, Tiruvannamalai, IND; 2 Community Medicine, Dhanalakshmi Srinivasan Medical College and Hospital, Siruvachur, IND

**Keywords:** allergic rhinitis (ar), eustachian tube dysfunction, hearing impairment, otoacoustic emissions, pure tone audiometry (pta), tympanometry

## Abstract

Background

Allergic rhinitis (AR) is a common chronic inflammatory disorder that can adversely affect both middle and inner ear function through Eustachian tube dysfunction (ETD) and cochlear involvement. Despite increasing evidence of auditory complications, hearing impairment among adults with AR remains under-recognized in routine clinical practice. This study aimed to estimate the prevalence of hearing impairment and identify sociodemographic and clinical factors independently associated with hearing impairment among adults with allergic rhinitis attending a tertiary care centre in Tiruvannamalai district of Tamil Nadu, India.

Materials and methods

A hospital-based cross-sectional study was conducted among 136 adults with clinically diagnosed allergic rhinitis attending the Otorhinolaryngology outpatient department of a tertiary care medical college and hospital in Tamil Nadu. Audiological assessment included pure-tone audiometry (PTA), tympanometry, and otoacoustic emissions (OAE). Data were analysed using IBM SPSS Statistics for Windows, version 26 (IBM Corp., Armonk, New York, United States). Descriptive statistics were expressed as frequencies, percentages, and mean ± SD. Independent samples t-test, one-way ANOVA, paired t-test, Pearson's correlation, and multivariable logistic regression were used to identify factors associated with hearing impairment. A p-value <0.05 was considered statistically significant.

Results

The mean age of the study participants was 32.51 ± 10.77 years, and 57.4% were female. Abnormal OAE findings, interpreted alongside tympanometry and PTA, were identified in 20 (14.7%) participants, suggesting possible cochlear dysfunction, while 28 (20.6%) had hearing impairment on PTA and 14 (10.3%) demonstrated Type C tympanograms indicating ETD in at least one ear. Mean PTA thresholds increased significantly with age, longer symptom duration, antihistamine use, congested nasal mucosa, abnormal OAE findings, and Type C tympanograms. Septal deviation showed an ear-specific association. In multivariable analysis, female sex was associated with lower odds of hearing impairment, whereas longer symptom duration and previous antihistamine use were associated with higher odds. Age and duration of symptoms also showed significant positive correlations with PTA thresholds.

Conclusion

Adults with allergic rhinitis demonstrated a considerable burden of audiological abnormalities. Female sex, prolonged symptom duration, and previous antihistamine use were independently associated with hearing impairment in the multivariable model. Routine audiological evaluation using PTA, tympanometry, and OAE should be considered in patients with persistent allergic rhinitis to facilitate early detection and appropriate management of auditory dysfunction.

## Introduction

Allergic rhinitis (AR) is a chronic IgE-mediated inflammatory disorder of the nasal mucosa triggered by environmental allergens such as pollen, dust mites, mold, and animal dander [1]. It is one of the most prevalent allergic conditions worldwide, affecting an estimated 10-30% of the population and contributing significantly to chronic respiratory disease burden [2]. The rising incidence of AR is linked to environmental and lifestyle factors including increased pollution, urbanization, reduced microbial exposure, and changing climate patterns [3]. Although often viewed as a benign upper airway disorder, AR has systemic effects on the respiratory tract, sleep, cognitive function, and overall quality of life [4].

AR is part of a broader atopic and inflammatory continuum involving both upper and lower airways [5]. The nasal cavity, paranasal sinuses, nasopharynx, Eustachian tube, and middle ear form an interconnected anatomical and physiological unit [6]. The “united airway” concept emphasizes shared mucosal characteristics, immunological mechanisms, and inflammatory pathways linking these regions [7]. Allergic inflammation in the nasal mucosa can extend to adjacent structures, disrupting their normal physiology and predisposing individuals to various ear-related symptoms and pathologies [8].

Audiological evaluation is essential for comprehensive auditory assessment and early detection of both conductive and sensorineural deficits [9]. Pure-tone audiometry (PTA) measures hearing thresholds across frequencies to identify hearing loss patterns. Tympanometry assesses middle ear compliance and pressure, providing insight into Eustachian tube function and middle ear pathology [10]. Otoacoustic emissions (OAE) testing evaluates outer hair cell integrity, serving as a sensitive indicator of early cochlear dysfunction. Combining these tests is particularly valuable in AR patients who may exhibit mixed or fluctuating hearing profiles depending on allergic inflammation severity and duration [11].

Despite growing research on AR and auditory dysfunction, many patients remain underdiagnosed or are evaluated only after significant symptoms develop. Ear-related symptoms are often attributed to temporary nasal congestion or seasonal allergies, leading to delayed hearing assessments [11]. This delay increases the risk of persistent hearing impairment, communication difficulties, reduced work efficiency, and diminished quality of life. Chronic, inadequately managed AR can cause repeated inflammatory episodes that cumulatively worsen middle and inner ear damage.

This hospital-based cross-sectional study was conducted in Tiruvannamalai district of Tamil Nadu, India, to estimate the prevalence of hearing impairment and identify sociodemographic and clinical factors independently associated with hearing impairment among adults with allergic rhinitis.

## Materials and methods

Study design and setting

This hospital-based cross-sectional study was carried out among adults diagnosed with AR who attended the outpatient department of Otorhinolaryngology at Arunai Medical College and Hospital, Tiruvannamalai, Tamil Nadu, India. The study was conducted over eight months between August 2024 and March 2025. The study was approved by the Institutional Ethics Committee of Arunai Medical College and Hospital (reference number: 046/2024), and written consent was obtained from the participants.

The diagnosis of AR was established based on the Indian Guidelines on AR by the Association of Otolaryngologists of India [12] as well as an Indian expert consensus by otolaryngologists [13], based on compatible nasal symptoms, relevant exposure history, and characteristic examination findings. The principal symptoms considered included sneezing, nasal itching, watery rhinorrhoea, and nasal congestion. Pale nasal mucosa and clear rhinorrhoea were considered supportive examination findings. Aeroallergen sensitization testing was not routinely performed because this study used a clinical diagnosis of AR.

Eligibility criteria

Patients aged 18-60 years who were medically diagnosed with AR using the above-mentioned criteria were considered eligible. The age range of 18-60 years was selected to focus on the adult AR population while limiting the potential contribution of advanced age-related hearing loss. The exclusion criteria were as follows: (i) patients with pre-existing ear diseases such as chronic suppurative otitis media, otosclerosis, or acute otitis media; (ii) patients with congenital ear deformities like anotia or microtia; (iii) patients using hearing aids; (iv) patients who were already medically diagnosed with sensorineural hearing loss; and (v) patients with incomplete clinical or audiological data.

Sampling method and sample size calculation

Participants were recruited using a convenience sampling approach from patients attending the Otorhinolaryngology outpatient department during the study period. Sample size estimation was based on the reported prevalence of sensorineural hearing loss among patients with AR (32%) from the study conducted by Sahni et al. at Patiala, Punjab, India [11]. Using the formula, $n = \frac{Z_{(1-\alpha/2)}^2 PQ}{d^2}$, where Z_(1-α/2)_= 1.96, P (prevalence)=32, Q (100-prevalence) =68, and d (absolute precision) = 8%. The minimum sample size with 95% confidence interval (CI) was calculated as 131. To compensate for possible incomplete records, 136 eligible participants were finally included in the analysis.

Data collection

Information was collected using a structured questionnaire (see Appendices) that had undergone pilot evaluation before the commencement of the study. Questionnaire development involved expert review followed by pilot testing among 30 patients who were subsequently excluded from the final analysis. Feedback obtained during the pilot phase was incorporated before preparing the final version. Internal consistency of the questionnaire was evaluated using Cronbach's alpha, which yielded a value of 0.71, indicating acceptable reliability.

The questionnaire comprised two sections. The first section documented sociodemographic characteristics, clinical history, and examination findings. History of antihistamine use was recorded as a binary variable based on participant-reported use of antihistamines before enrolment. The treatment variable captured previous antihistamine use but did not systematically quantify drug type, dose, duration or adherence. The second section recorded the audiological assessment results performed according to standardized departmental protocols. Information regarding occupational/recreational noise exposure, ototoxic medication exposure, diabetes mellitus and hypertension was not systematically collected. The severity of AR was also not formally graded using a validated symptom-severity or disease-control scale.

Audiological assessment

All enrolled participants underwent a comprehensive audiological evaluation comprising (a) OAE, (b) tympanometry, and (c) PTA. All the procedures were conducted in a soundproof booth certified under International Organization for Standardization (ISO) standards.

OAE was performed to assess the function of outer hair cells in the cochlea, which is essential for identifying early cochlear involvement. The OAE was performed at 2000 Hz, 3000 Hz, 4000 Hz, and 6000 Hz after making sure that the external ear canal is free of debris or cerumen [14]. The results of OAE included: (i) Pass: Presence of clear, repeatable emissions across test frequencies, which suggests normal cochlear outer hair cell function; (ii) Refer: Poor or absent emissions, which indicate possible cochlear dysfunction. OAE findings can be altered by Eustachian tube dysfunction (ETD) and secretory otitis media. OAE findings were interpreted alongside tympanometry and pure-tone audiometric findings and were not considered diagnostic of sensorineural hearing loss in isolation [14]. OAE was conducted using an Audio-SMART model (Neurosoft, Ivanovo, Russia).

Tympanometry was used to evaluate the condition of the middle ear and to ensure that conductive abnormalities did not confound the identification of sensorineural deficits [15]. The results of tympanometry included: (i) Type A: Normal compliance at atmospheric pressure, which indicates normal middle ear function; (ii) Type B: Flat tracing, which indicates reduced mobility of the tympanic membrane (fluid or pathology); (iii) Type C: Negative pressure peak, which indicates ETD [15]. However, no Type B tympanograms were observed in this study dataset. Tympanometry was conducted using the MT 10 portable screening tympanometer (Interacoustics, Middelfart, Denmark).

PTA served as the primary tool for identifying the degree and type of hearing loss with particular emphasis on detecting sensorineural patterns. Air- and bone-conduction thresholds were assessed according to standard audiometric procedures. Air-conduction thresholds were measured at 500, 1000, 2000, 4000, 6000 and 8000 Hz, while bone-conduction thresholds were assessed at the relevant frequencies [16]. The results of PTA included: (i) Normal Hearing: 0-25 dB; (ii) Mild impairment: 26-40 dB; (iii) Moderate impairment: 41-60 dB; (iv) Severe impairment: 61-80 dB; (v) Profound impairment including deafness: ≥ 81 dB [17]. Hearing loss was classified as conductive, sensorineural, or mixed based on the presence or absence of an air-bone gap in conjunction with the audiometric configuration [18]. PTA was conducted using an Equinox model (Interacoustics).

Hearing impairment was defined based on the four-frequency pure-tone average, calculated as the mean hearing threshold at 500 Hz, 1000 Hz, 2000 Hz, and 4000 Hz. A PTA of ≥25 dB was considered hearing impairment, which included conductive, sensorineural, as well as mixed hearing loss [19]. For the primary outcome, participants were classified as having hearing impairment if the criterion was met in either ear.

Statistical analysis

The data collected were entered into Microsoft Excel (Microsoft Corporation, Redmond, Washington, United States), numerically coded, and analyzed using IBM SPSS Statistics for Windows, version 26 (Released 2018; IBM Corp., Armonk, New York, United States). Continuous variables are presented as mean ± standard deviation (SD), whereas categorical variables are summarized using frequencies and percentages. The Kolmogorov-Smirnov test was applied to evaluate the normality of data distribution. Depending on the variable under investigation, comparisons between groups were performed using the independent samples t-test, one-way analysis of variance (ANOVA), or paired t-test. Pearson's correlation coefficient was calculated to examine the relationship between age, duration of AR, and PTA thresholds.

Variables with p<0.20 in bivariate analysis, together with clinically relevant covariates identified a priori, were considered for multivariable logistic regression. Model calibration was assessed using the Hosmer-Lemeshow goodness-of-fit test (χ² = 5.56, p = 0.29). The overall model was statistically significant (χ² = 44.08, p<0.001), explained 40.2% of the observed variation according to the Nagelkerke R² statistic, and correctly classified 80.9% of study participants. Statistical significance was defined as a two-sided p-value <0.05.

## Results

A total of 136 adults with AR were included in the final analysis. The participants had a mean age of 32.51 ± 10.77 years (range, 18-60 years). Individuals aged 26-40 years constituted the largest age group (39.7%), followed by those aged 18-25 years (36.8%). Female sex accounted for 57.4% of the study population, while approximately two-thirds of the participants were residents of urban areas (66.9%). The basic characteristics of the study participants are summarized in Table 1.

**Table 1:** Socio-clinical characteristics of the study participants (N=136)

Characteristics		Frequency	Percentage
Age (years)	18-25	50	36.8
	26-40	54	39.7
	>40	32	23.5
Gender	Male	58	42.6
	Female	78	57.4
Residence	Urban	91	66.9
	Rural	45	33.1
Duration of allergic rhinitis (years)	<2	26	19.1
	2-5	92	67.6
	>5	18	13.2
History of taking antihistamines	Yes	81	59.6
	No	55	40.4
Nasal septum	Midline	61	44.9
	Deviated to right	42	30.9
	Deviated to left	33	24.3
Nasal mucosa	Normal	47	34.6
	Congested	35	25.7
	Pale	54	39.7

Audiological assessment

OAE testing identified evidence suggestive of cochlear dysfunction in a minor proportion of participants. Overall, 20 patients (14.71%; 95% CI: 9.73-21.63) demonstrated a "Refer" result in at least one ear, indicating possible cochlear dysfunction, whereas the remaining participants had normal OAE findings (Figure 1).

**Figure 1:**
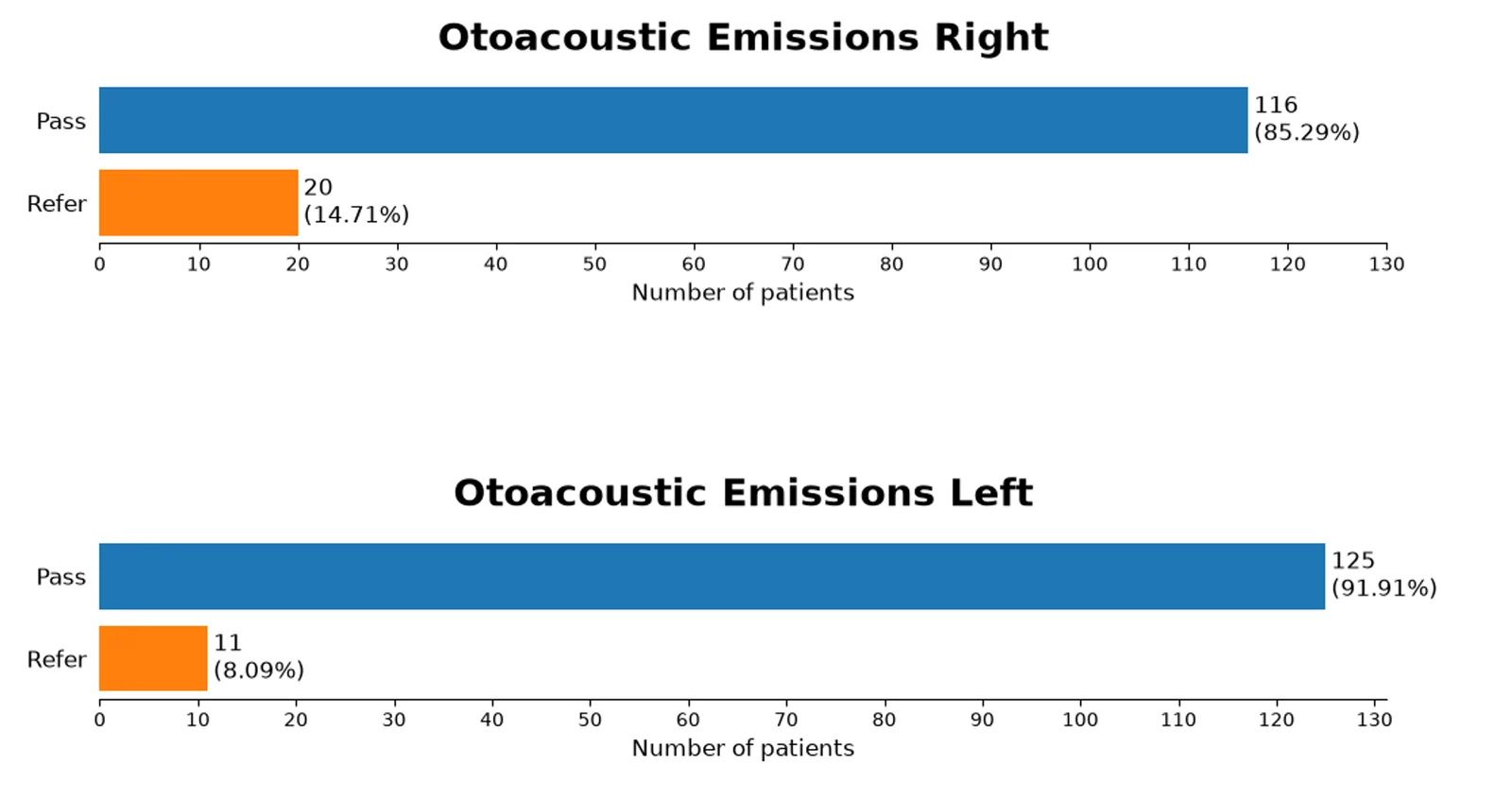
Results of otoacoustic emissions

Tympanometry evaluation showed predominantly normal middle-ear function. Nevertheless, 14 participants (10.29%; 95% CI: 6.23-16.54) exhibited Type C tympanograms, consistent with ETD in one or both ears (Figure 2).

**Figure 2:**
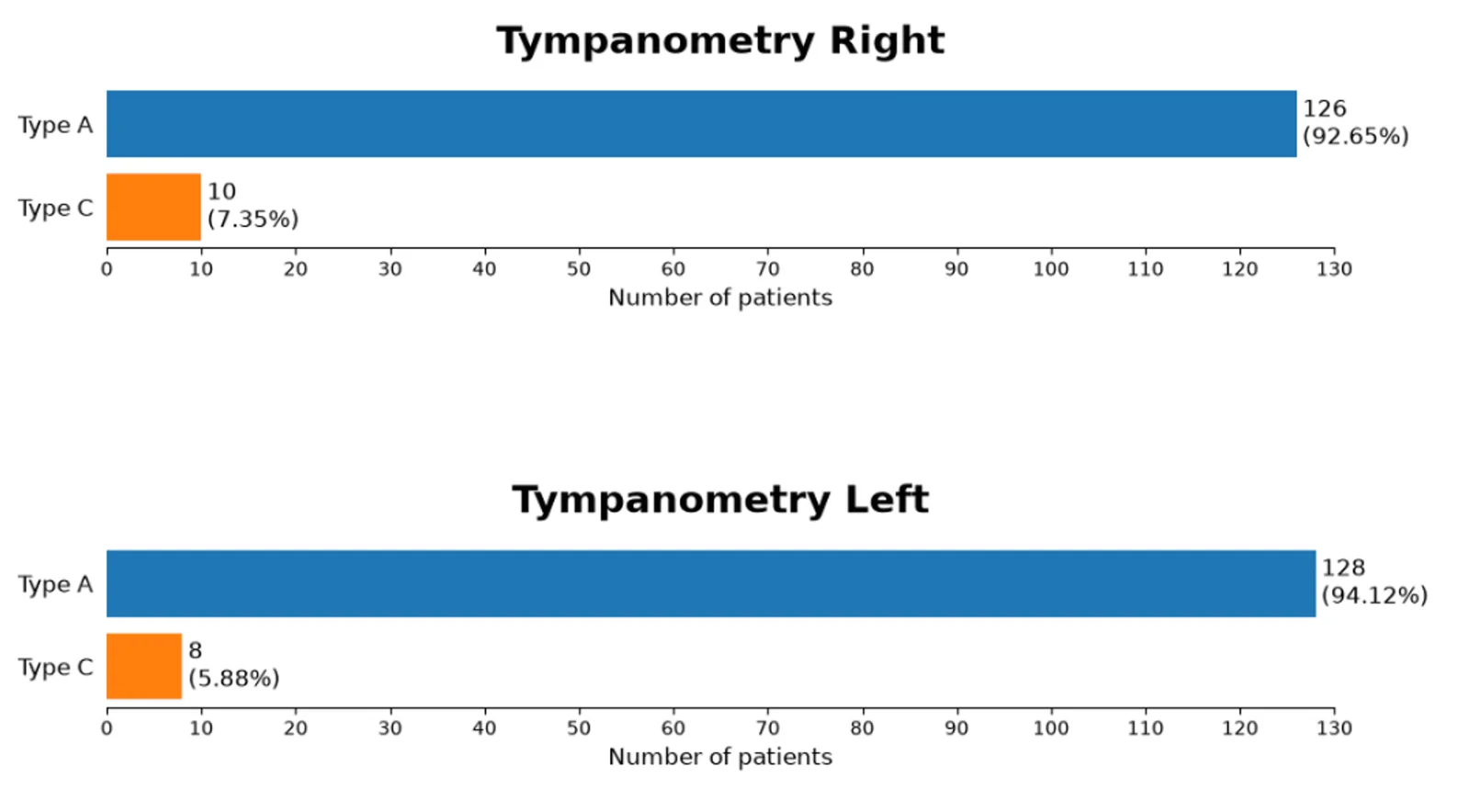
Results of tympanometry

PTA revealed that 28 participants (20.6%; 95% CI: 14.65-28.15) had hearing impairment affecting at least one ear. Of these 28 participants, the majority (n=20, 71.4%) had sensorineural hearing loss (SNHL), while five (17.8%) had conductive hearing loss and three (10.7%) had mixed hearing loss. Of the 20 participants who had sensorineural hearing loss, seven had mild SNHL while 13 had moderate SNHL. All five participants who had conductive hearing loss were in the mild category (Figure 3).

**Figure 3:**
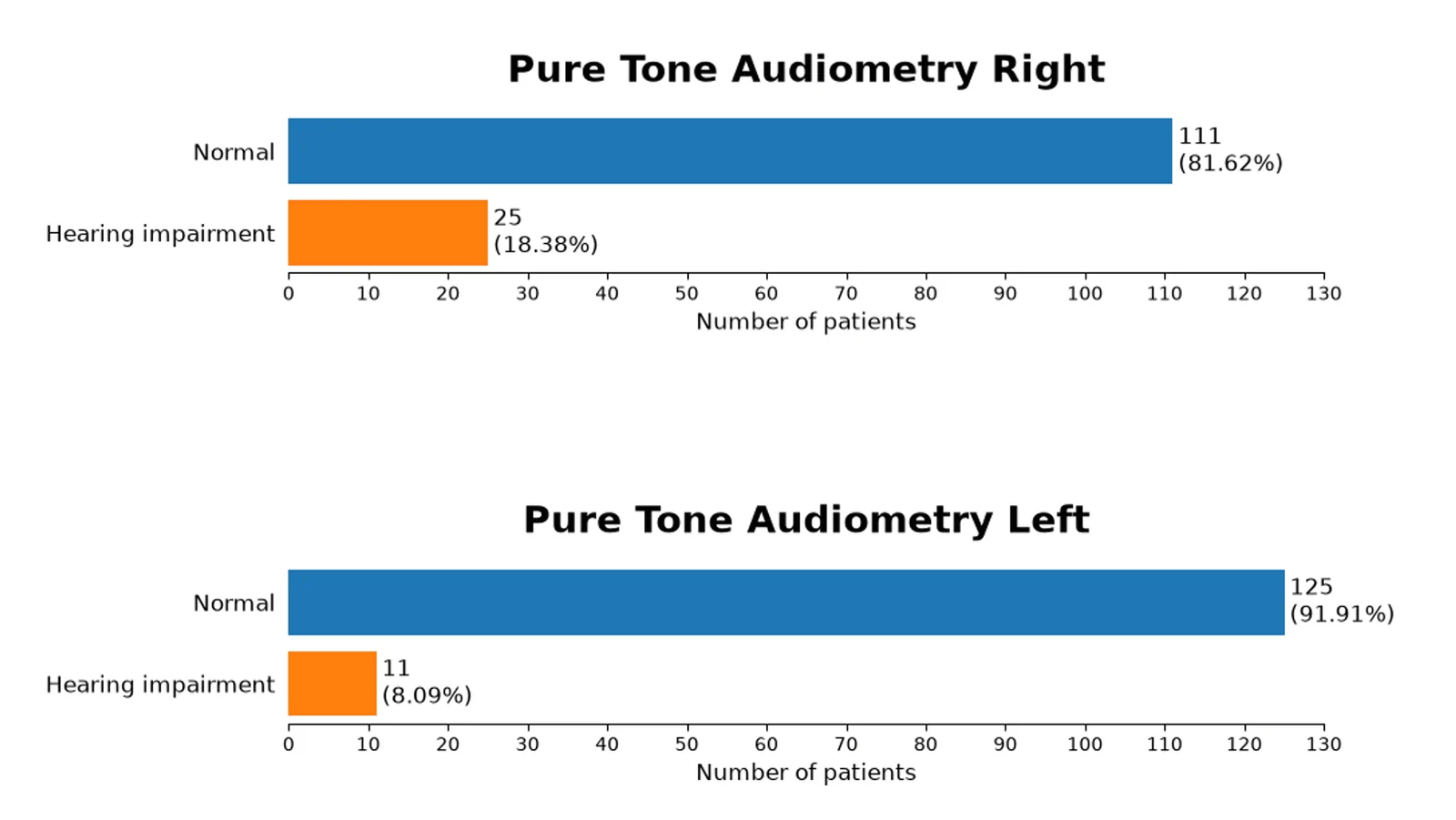
Results of pure-tone audiometry

The mean PTA threshold of the right ear increased significantly with advancing age (p-value = 0.01), with participants aged >40 years demonstrating significantly higher hearing thresholds than those aged 18-25 years. A progressive increase in mean PTA was also observed with increasing duration of AR (p-value = 0.03), and post hoc analysis revealed significantly higher thresholds among participants with symptom duration of two to five years and >5 years compared with those having symptoms for <2 years. Participants receiving antihistamine treatment had significantly higher mean PTA thresholds than those not receiving treatment (p-value = 0.03). Mean PTA also varied significantly according to nasal septal deviation (p-value = 0.04) and nasal mucosal status (p-value = 0.02), with higher thresholds observed among patients with septal deviation and congested nasal mucosa. No statistically significant difference in mean PTA was observed between male and female patients (Table 2).

**Table 2:** Variation of mean pure-tone audiometry (Right) among study participants based on specific characteristics (N=136) *p-value <0.05 is statistically significant; ^Independent samples t-test was used; ^#^One-way ANOVA was used; ^†^Statistically significant difference in post hoc Tukey test, Age (in years): >40 – 18 to 25 (mean difference 6.11); Duration of symptoms: 2-5 to <2 (mean difference 9.34), >5 to 2-5 (mean difference 5.16), >5 to <2 (mean difference 14.51); Nasal septum: Deviated to right – Midline (mean difference 5.13), Deviated to left – Midline (mean difference 7.50); Nasal mucosa: Congested-Normal (mean difference 8.24), Congested-Pale (mean difference 5.80)

Characteristics		PTA (dB), mean ±SD	Table value	p-value
Age (in years)^ #^	18-25	20.19 ±7.46	6.13	0.01*
	26-40	22.46 ±7.92		
	>40^†^	26.31 ±7.77		
Gender^^^	Male	23.54 ±8.40	2.5	0.11
	Female	21.80 ±7.68		
Duration of symptoms (in years)^ #^	< 2^†^	14.30 ±1.65	8.36	0.03*
	2-5^†^	23.64 ±7.53		
	>5^†^	28.81 ±7.03		
Antihistamines taken^^^	Yes	26.04 ±7.38	4.52	0.03*
	No	17.43 ±5.89		
Nasal septum^#^	Midline	19.10 ±7.46	7.77	0.04*
	Deviated to right^†^	24.23 ±7.35		
	Deviated to left^†^	26.60 ±7.32		
Nasal mucosa^#^	Normal	19.43 ±7.43	9.86	0.02*
	Congested^†^	27.68 ±6.11		
	Pale	21.88 ±8.06		

The mean PTA threshold of the left ear demonstrated a significant increase with advancing age (p-value = 0.01), with participants aged above 40 years exhibiting significantly higher hearing thresholds than younger individuals. Female participants had significantly higher mean PTA thresholds than males (p-value = 0.01). Mean hearing thresholds also increased significantly with longer duration of AR (p-value = 0.01), with post hoc analysis demonstrating significant differences between all duration categories. Participants receiving antihistamine treatment had significantly higher mean PTA thresholds than those not receiving treatment (p-value = 0.04). Nasal mucosal status was significantly associated with hearing thresholds (p-value = 0.01), with congested nasal mucosa demonstrating the highest mean PTA values. Although higher mean thresholds were observed among participants with nasal septal deviation, the association was not statistically significant (Table 3).

**Table 3:** Variation of mean pure-tone audiometry (Left) among study participants based on specific characteristics (N=136) *p-value <0.05 is statistically significant; ^Independent samples t-test was used; ^#^One-way ANOVA was used; ^†^Statistically significant difference in post hoc Tukey test Age (in years): >40 to 18-25 (mean difference 5.81); Duration of symptoms: 2-5 to <2 (mean difference 9.33), >5 to 2-5 (mean difference 4.84), >5 to <2 (mean difference 14.18); Nasal septum: Deviated to right – Midline (mean difference 5.25), Deviated to left – Midline (mean difference 7.20); Nasal mucosa: Congested-Normal (mean difference 8.19), Congested-Pale (mean difference 5.17)

Characteristics		PTA (dB), mean±SD	Table value	p-value
Age (years)^#^	18-25	20.92±7.31	5.63	0.01*
	26-40	22.89±7.98		
	>40^†^	26.73±7.66		
Gender^^^	Male	23.92±8.61	7.61	0.01*
	Female	22.43±7.36		
Duration of symptoms (years)^ #^	< 2^†^	14.89±1.90	11.89	0.01*
	2-5^†^	24.22±7.41		
	>5^†^	29.07±7.16		
Antihistamines taken^^^	Yes	26.44±7.29	4.28	0.04*
	No	18.16±6.06		
Nasal septum^#^	Midline	19.73±7.61	2.35	0.10
	Deviated to right^†^	24.98±6.91		
	Deviated to left^†^	26.93±7.34		
Nasal mucosa^#^	Normal	19.76±7.45	12.17	0.01*
	Congested^†^	27.95±5.98		
	Pale	22.78±7.96		

Participants with Type C tympanograms demonstrated significantly higher mean PTA thresholds than those with Type A tympanograms in both ears (p-value < 0.001). In the right ear, the mean PTA threshold was 29.35 ± 2.56 dB among participants with Type C tympanograms compared with 21.89 ± 8.09 dB among those with Type A tympanograms. Similarly, in the left ear, participants with Type C tympanograms had significantly higher mean PTA thresholds than those with Type A tympanograms, indicating an association between ETD and poorer hearing thresholds (Table 4).

**Table 4:** Comparison of mean pure-tone audiometry thresholds according to tympanometry findings (N=136) *p value<0.05 is statistically significant; Independent samples t-test was used

Ear	Tympanogram	Frequency (n)	PTA (dB), mean±SD	t value	p value
Right	Type A	126	21.89±8.09	-2.89	<0.001*
	Type C	10	29.35±2.56		
Left	Type A	128	22.53±7.98	-2.64	<0.001*
	Type C	8	30.06±2.75		

Participants with abnormal OAE findings ("Refer") demonstrated significantly higher mean PTA thresholds than those with normal OAE findings ("Pass") in both ears. In the right ear, the mean PTA threshold was significantly higher among participants with abnormal OAE findings (29.61 ± 4.16 dB) than among those with normal OAE findings (17.42 ± 6.06 dB; p = 0.04). Similarly, in the left ear, participants with abnormal OAE findings exhibited significantly higher mean PTA thresholds than those with normal OAE findings (30.15 ± 5.82 dB vs. 22.02 ± 7.74 dB; p <0.001), suggesting a close association between cochlear dysfunction and elevated hearing thresholds (Table 5).

**Table 5:** Comparison of mean pure-tone audiometry thresholds according to otoacoustic emissions findings (N=136) *p value<0.05 is statistically significant; Independent samples t-test was used

Ear	OAE Result	n	PTA (dB), mean±SD	t value	p value
Right	Pass	116	17.42±6.06	-11.21	<0.001*
	Refer	20	29.61±4.16		
Left	Pass	125	22.02±7.74	-4.31	<0.001*
	Refer	11	30.15±5.82		

Pearson's correlation analysis demonstrated a statistically significant positive relationship between participant age and PTA thresholds (r = 0.228, p = 0.008). Duration of AR also showed a moderate positive correlation with hearing thresholds (r = 0.562, p < 0.001), indicating that prolonged disease duration was associated with progressively poorer auditory function.

Multivariable logistic regression analysis identified sex, duration of AR, and previous antihistamine treatment as independently associated with hearing impairment. Female participants had lower odds of hearing impairment compared with male participants (adjusted OR= 0.22; 95% CI 0.07-0.67). Compared with participants reporting symptoms for less than two years, those with symptom duration of 2-5 years and more than five years had higher odds of hearing impairment (adjusted OR = 1.21; 95% CI 1.03-2.68 and adjusted OR = 1.99; 95% CI 1.52-2.98, respectively). Participants who reported a history of previous antihistamine treatment had lower odds of hearing impairment than those who reported antihistamine use (adjusted OR = 0.15; 95% CI 0.05-0.83). Age, nasal septal deviation, and nasal mucosal status were not independently associated with hearing impairment after adjustment (Table 6).

**Table 6:** Independent predictors of hearing impairment as per pure tone audiometry findings (N=136) *p value < 0.05 is statistically significant; multivariable logistic regression was used

Variables	Hearing impairment in any one ear		Unadjusted OR (95% CI)	Adjusted OR (95% CI)
	Yes n (%)	No n (%)		
Age (in years)				
18 to 25	6 (12)	44 (88)	1	1
26 to 40	12 (22.2)	42 (77.8)	2.09 (0.72-6.09)	1.35 (0.37-4.86)
>40	10 (31.3)	22 (68.8)	3.33 (1.07-10.36)*	1.18 (0.27-5.13)
Gender				
Male	18 (31)	40 (69)	1	1
Female	10 (12.8)	68 (87.2)	0.32 (0.13-0.77)*	0.22 (0.07-0.67)*
Duration of symptoms (in years)				
<2	6 (23.1)	20 (76.9)	1	1
2 to 5	18 (19.6)	74 (80.4)	0.81 (0.28-2.31)	1.21 (1.03-2.68)*
>5	10 (55.6)	8 (44.4)	4.17 (1.13-15.33)*	1.99 (1.52-2.98)*
Antihistamine use				
Yes	26 (32.1)	55 (67.9)	1	1
No	2 (3.6)	53 (96.4)	0.08 (0.01-0.35)*	0.15 (0.05-0.83)*
Nasal septum				
Midline	9 (14.8)	52 (85.2)	1	1
Deviated to right	8 (19)	34 (81)	1.35 (0.47-3.86)	0.93 (0.22-3.95)
Deviated to left	11 (33.3)	22 (66.7)	2.88 (1.05-7.94)*	1.08 (0.21-5.15)
Nasal mucosa				
Normal	6 (12.8)	41 (87.2)	1	1
Congested	12 (34.3)	23 (65.7)	3.56 (1.18-10.76)*	0.50 (0.08-3.19)
Pale	10 (18.5)	44 (81.5)	1.55 (0.51-4.65)	0.49 (0.11-2.13)

## Discussion

This study provides an overview of hearing impairment among adults with AR attending a tertiary care centre in Tiruvannamalai district, Tamil Nadu, India. It also sheds light on the proportion of hearing impairment among adults with AR.

Sex was associated with audiometric findings in this study, although the pattern differed according to the analytical approach and ear assessed. Female participants had significantly higher mean PTA thresholds in the left ear, whereas no significant sex difference was observed in the right ear. In the multivariable analysis of hearing impairment affecting either ear, however, female sex was associated with lower odds of hearing impairment. A similar study conducted in Andhra Pradesh, India, by Reddy et al. reported no statistical significance between sex and hearing impairment among patients with AR [20]. However, a gradual increase in hearing impairment was reported in that study, which was not found to be statistically significant in this study. The discrepancy between these findings and the present study may reflect differences in sample characteristics, outcome definitions, audiometric assessment, and statistical methods. Given the inconsistent ear-specific findings in the present study, the observed association with sex should be interpreted cautiously.

In this study, 14.7% of the study participants had abnormal OAE in either one of their ears, and 71.4% of the participants who had hearing impairment had sensorineural hearing loss. In comparison, 28% of the study participants with AR exhibited varying degrees of OAE as per Choudhary from Punjab, India [21], and only 19.5% of the study participants exhibited mild SNHL according to Reddy et al. from Andhra Pradesh [20]. Chronic inflammatory changes, longstanding nasal mucosal edema, and persistent ETD as part of AR may contribute to middle-ear ventilation problems and conductive deficits [22]. Simultaneously, inflammatory mediators (histamine, leukotrienes, and cytokines) have been proposed to influence cochlear microcirculation and outer-hair-cell function, which could explain the abnormal OAE findings and a high proportion of sensorineural hearing loss in this study [23]. Gao et al. from China have reported that AR could lead to hearing impairment manifested as hidden hearing loss [24]. Direct comparison with the present findings should be made cautiously because these studies included both air- and bone-conduction testing, whereas the current study was a single-group cross-sectional study. Differences in age distribution, outcome definitions and audiometric classification may also have contributed to the observed variation in prevalence. 

Duration of symptoms was found to be significantly associated with hearing impairment in this study. According to Ankle et al., the correlation between AR chronicity and hearing loss was also statistically significant [25]. The most common type of impedance curve seen in this study was Type A. This finding is not unexpected because allergic rhinitis does not invariably result in ETD. Type C tympanograms reflect negative middle-ear pressure and represent one manifestation of ETD rather than an obligatory consequence of allergic rhinitis. The predominance of Type A curves therefore suggests that middle-ear pressure abnormalities were present only in a subset of participants. This was consistent with the findings of a similar study conducted by Prabakaran et al. from Tamil Nadu, India, where Type A was also the most frequent impedance pattern among patients with allergic rhinitis [26]. This suggests that although ETD may occur in AR, it is not present in all affected individuals. A case-control study of persistent AR by Mukherjee et al. reported that Type A curves remained common even among patients with persistent AR, although abnormal tympanograms increased with disease severity [27].

In this study, participants who reported no previous antihistamine treatment had lower odds of hearing impairment than those who reported treatment. This finding may also reflect confounding by indication, as patients with more persistent or severe allergic symptoms may have been more likely to receive antihistamines. The available evidence regarding the effect of antihistamine therapy on ETD and auditory outcomes remains inconclusive. A review by Juszczak and Loftus reported an association between AR and ETD but noted that studies evaluating oral antihistamines and intranasal corticosteroids had not demonstrated consistent symptomatic benefit for ETD [28]. Conversely, a prospective study by Ma et al. reported improvement in Eustachian tube function after one month of combined intranasal mometasone and oral loratadine treatment among patients with house dust mite-related AR [29]. These differences highlight the uncertainty surrounding the effect of antihistamine treatment on auditory outcomes and indicate that the treatment association observed in this study should be considered hypothesis-generating rather than causal.

The association between septal deviation and PTA was statistically significant for the right ear but not the left ear in this study. This asymmetry should be interpreted cautiously. Septal deviation is anatomically asymmetric and may influence nasal airflow and Eustachian tube ventilation differently on either side. However, the present cross-sectional design and relatively small number of participants with hearing impairment do not permit a definitive explanation, and the ear-specific finding may also reflect sampling variability.

This study reported a statistically significant positive correlation between age of the study participants, duration of symptoms, and the PTA findings. On the contrary, Karabulut et al. from Turkey reported that the patients with AR had better hearing than the control group at 8000 Hz when PTA was performed [30]. Age was associated with continuous PTA thresholds in the bivariate analysis but did not remain statistically significant in the multivariable model for the binary hearing-impairment outcome. This difference may reflect the distinction between the continuous and dichotomized outcomes, adjustment for correlated variables such as disease duration, and limited statistical power resulting from the small number of participants with hearing impairment. Therefore, the absence of statistical significance in the adjusted model should not be interpreted as evidence that age has no relationship with hearing thresholds.

Strengths and limitations

A major strength of this study was the use of standardized audiological assessment incorporating PTA, tympanometry and OAE, together with systematic collection of demographic and clinical information using a pilot-tested structured questionnaire with acceptable internal consistency. The use of standardized data collection procedures further improved the reliability and consistency of the information obtained.

Several limitations warrant consideration while interpreting the study findings. Participants were recruited exclusively from one tertiary care centre, which may restrict the external validity of the results. Objective allergen sensitization testing (including serum-specific IgE or skin-prick testing) was not performed, and therefore the study could not characterize allergen-specific phenotypes of allergic rhinitis. Only a small number of participants met the hearing-impairment outcome, limiting the complexity and precision of multivariable regression estimates. As previous antihistamine use was recorded only as a binary variable, information regarding the specific drug, dose, duration, adherence, and treatment indication was unavailable. Consequently, the observed association between previous antihistamine use and hearing impairment may be influenced by confounding by indication and should not be interpreted as a treatment effect. Although the upper age limit of 60 years reduced the contribution of more advanced age-related hearing loss, residual confounding by age-related auditory changes cannot be excluded. Residual confounding is possible because potentially relevant causes of hearing impairment, including noise exposure, ototoxic medication use, diabetes and hypertension, were not systematically assessed in this study. Furthermore, the use of a convenience sampling strategy could have resulted in selection bias. Finally, the cross-sectional design limits the ability to infer causality or determine the temporal sequence between the identified independent factors and hearing impairment.

## Conclusions

AR was associated with a substantial burden of auditory dysfunction, with a considerable proportion of patients demonstrating probable cochlear dysfunction on audiological evaluation. Longer duration of symptoms, previous antihistamine use, and female sex were independently associated with hearing impairment, while Type C tympanograms and abnormal otoacoustic emission findings were associated with significantly higher PTA thresholds. These findings highlight the importance of comprehensive audiological assessment, including PTA, tympanometry, and otoacoustic emissions, in adults with AR, particularly those with persistent symptoms. Hence, routine audiological screening should be considered in adults with AR, especially those with prolonged disease duration or additional clinical risk factors, to facilitate early detection and appropriate management of hearing impairment. Prospective studies are required to determine whether effective control of AR modifies subsequent auditory outcomes.

## Figures and Tables

**Figure 4:**
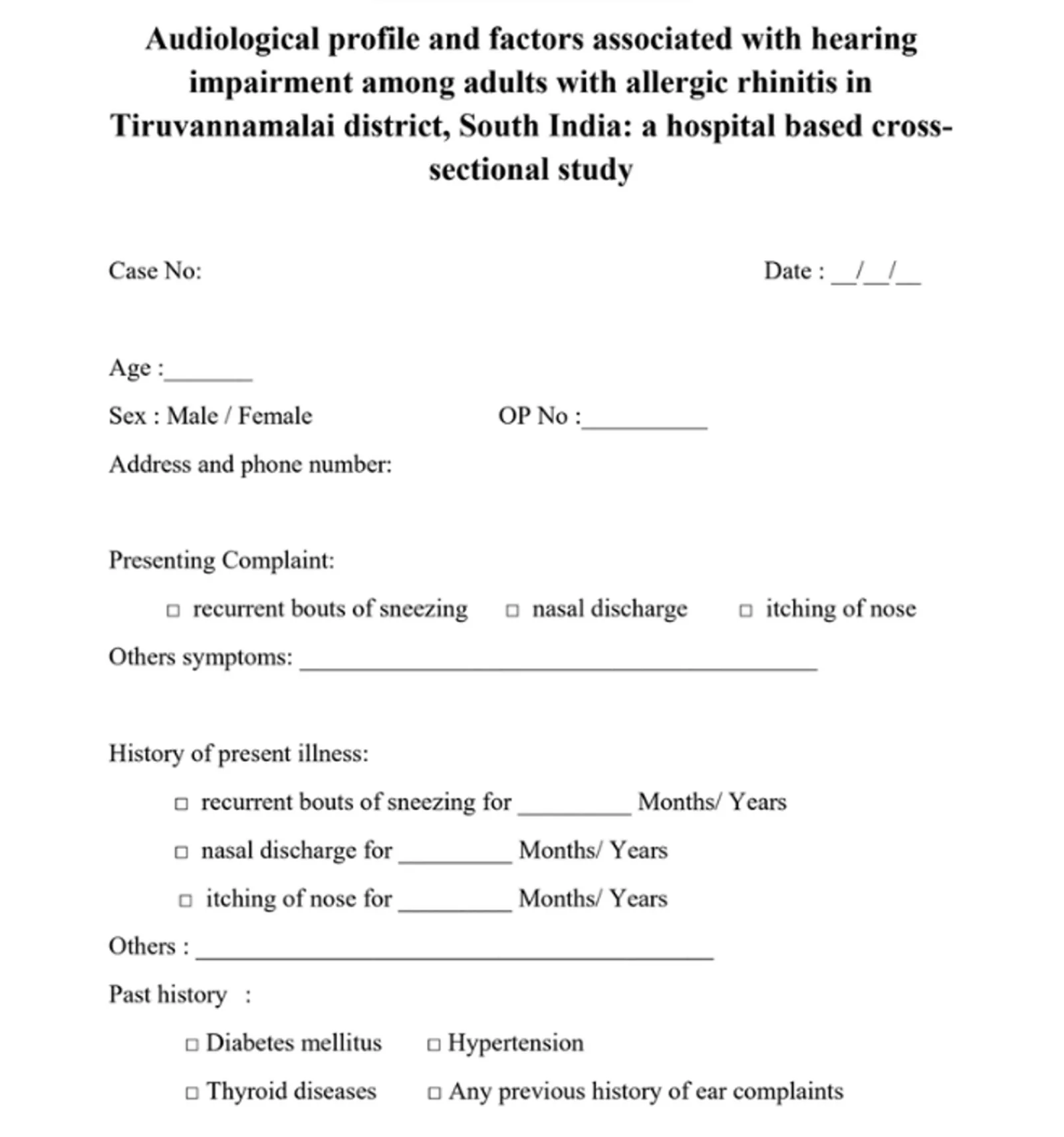
Study questionnaire, page 1

**Figure 5:**
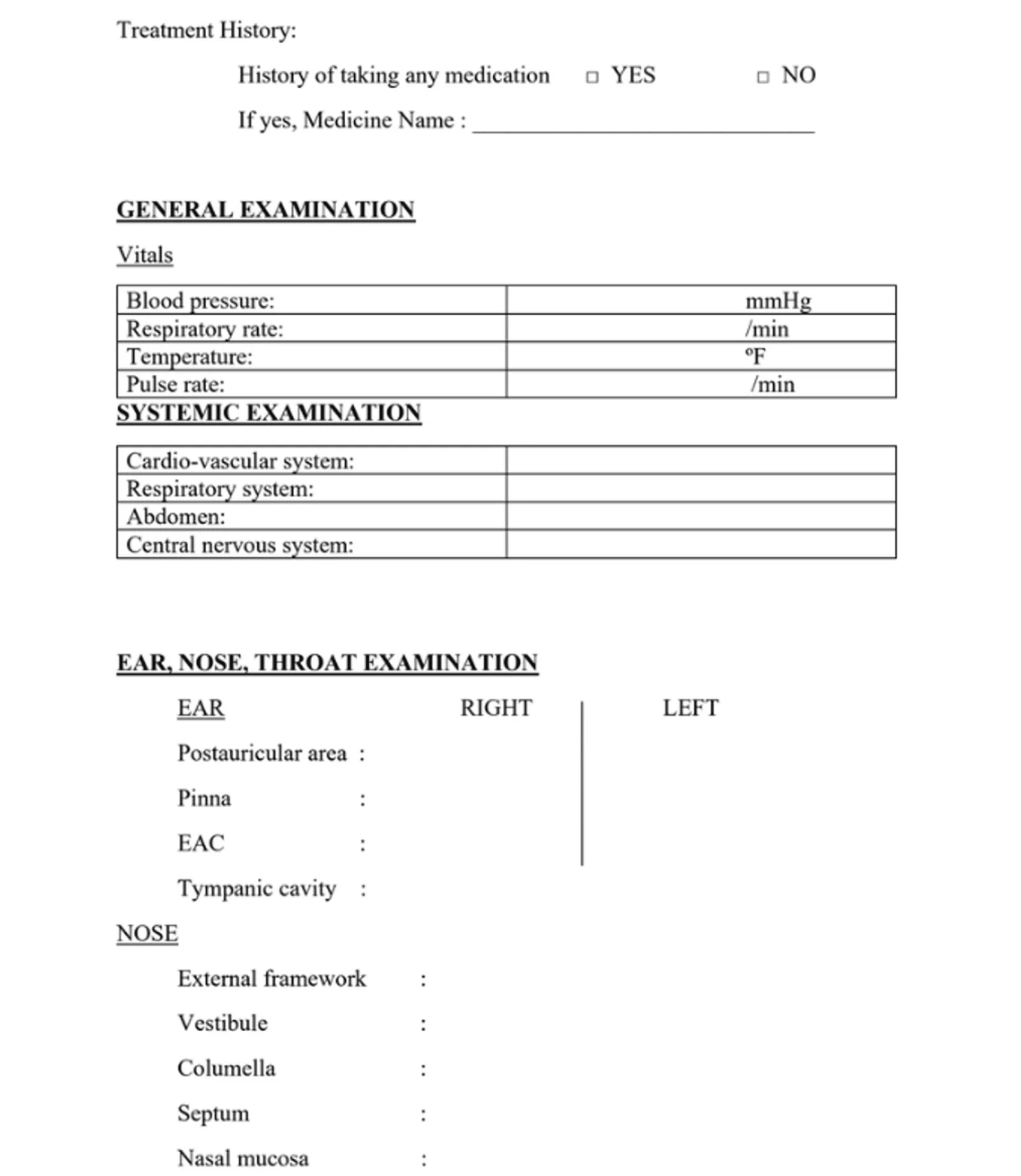
Study questionnaire, page 2

**Figure 6:**
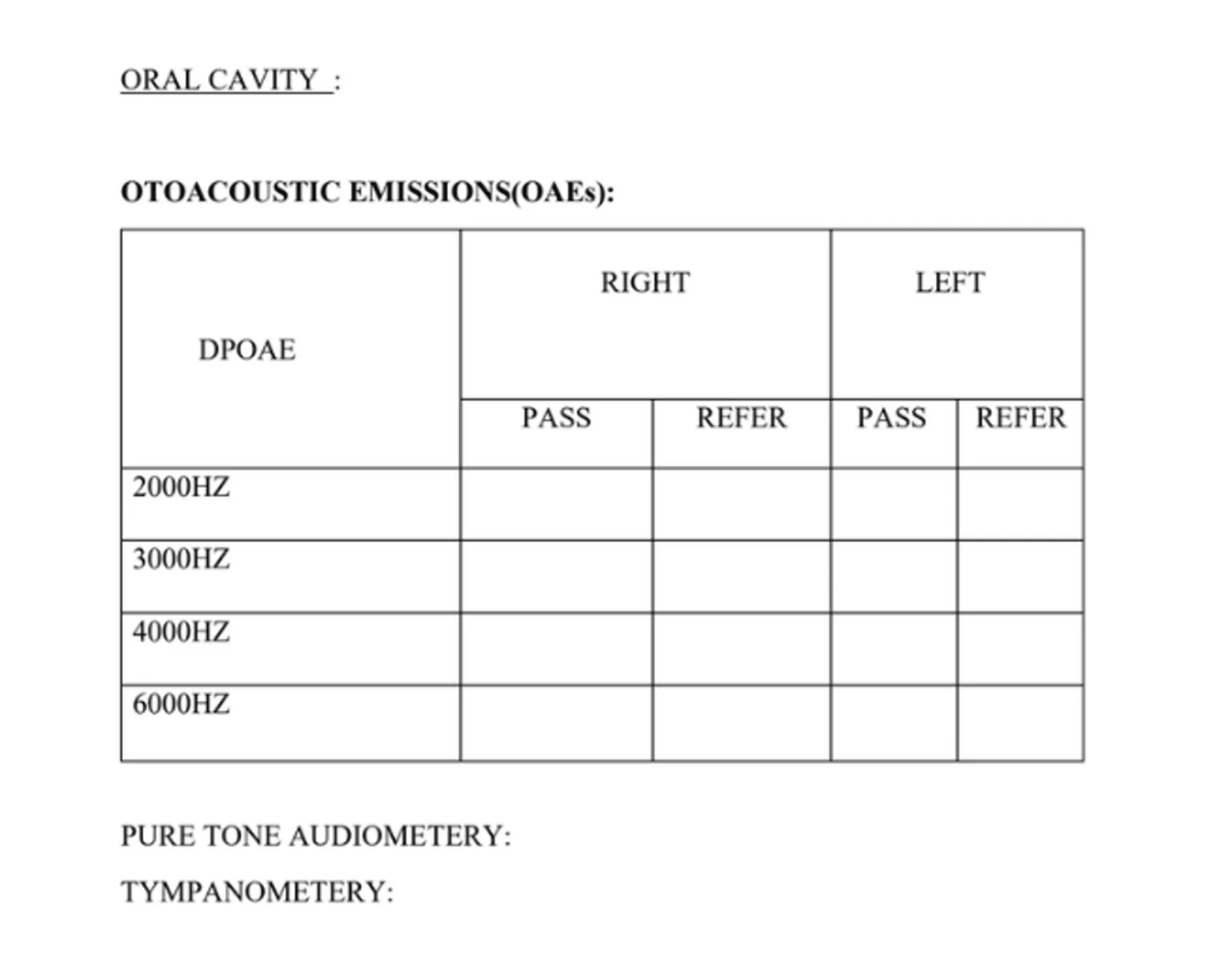
Study questionnaire, page 3

## Appendices

Questionnaire 
